# Intravaginal immunisation using a novel antigen-releasing ring device elicits robust vaccine antigen-specific systemic and mucosal humoral immune responses

**DOI:** 10.1016/j.jconrel.2017.01.018

**Published:** 2017-03-10

**Authors:** Paul F. McKay, Jamie F.S. Mann, Aditya Pattani, Vicky Kett, Yoann Aldon, Deborah King, R. Karl Malcolm, Robin J. Shattock

**Affiliations:** a Imperial College London, Department of Medicine, Division of Infectious Diseases, Section of Virology, Norfolk Place, London W2 1PG, UK; b School of Pharmacy, Queen's University Belfast, 97 Lisburn Road, Belfast BT9 7BL, UK

## Abstract

The generation of effective levels of antigen-specific immunity at the mucosal sites of pathogen entry is a key goal for vaccinologists. We explored topical vaginal application as an approach to initiate local antigen-specific immunity, enhance previously existing systemic immunity or re-target responses to the mucosae. To deliver a protein vaccine formulation to the vaginal mucosal surface, we used a novel vaginal ring device comprising a silicone elastomer body into which three freeze-dried, rod-shaped, hydroxypropylmethylcellulose inserts were incorporated. Each rod contained recombinant HIV-1 CN54gp140 protein (167 μg) ± R848 (167 μg) adjuvant. The inserts were loaded into cavities within each ring such that only the ends of the inserts were initially exposed.

Sheep received a prime-boost vaccination regime comprising intramuscular injection of 100 μg CN54gp140 + 200 μg R848 followed by three successive ring applications of one week duration and separated by one month intervals. Other sheep received only the ring devices without intramuscular priming. Serum and vaginal mucosal fluids were sampled every two weeks and analysed by CN54gp140 ELISA and antigen-specific B cells were measured by flow cytometry at necropsy. Vaccine antigen-specific serum antibody responses were detected in both the intramuscularly-primed and vaginal mucosally-primed groups. Those animals that received only vaginal vaccinations had identical IgG but superior IgA responses. Analysis revealed that all animals exhibited mucosal antigen-specific IgG and IgA with the IgA responses 30-fold greater than systemic levels. Importantly, very high numbers of antigen-specific B cells were detected in local genital draining lymph nodes.

We have elicited local genital antigen-specific immune responses after topical application of an adjuvanted antigen formulation within a novel vaginal ring vaccine release device. This regimen and delivery method elicited high levels of antigen-specific mucosal IgA and large numbers of local antigen-reactive B cells, both likely essential for effective mucosal protection.

## Graphical abstract

**Figure:**
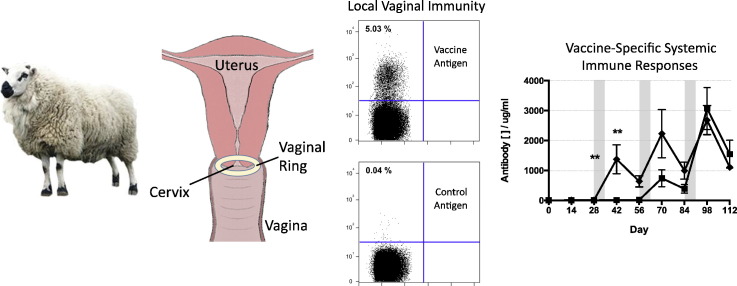

## 1 Introduction

While it is relatively easy to elicit antigen-specific serum antibodies, it is much more difficult to establish meaningful levels of specific antibodies at mucosal surfaces, the major route of viral invasion. In this study, we sought to determine if mucosal vaccination using topical vaginal application could initiate local antigen-specific immunity, and/or enhance or re-target previously existing systemic immunity to the mucosae. Previous studies in mice have shown that vaccines generating high levels of systemic antigen-specific immunity can lead to the appearance of genital tract mucosal surface responses, derived from the systemic compartment [1], [2], [3]. Also in mice, studies have shown that direct mucosal vaccination via the intranasal, sublingual or intravaginal routes can efficiently enhance immune responses at other mucosal surfaces. This observation is generally attributed to common mucosal linkage, but this distribution of mucosal associated responses has been found to be very weak or absent in larger animals, and particularly man [4], [5], [6], [7]. In an effort to generate local mucosal immunity, a number of researchers have explored genital tract targeted vaccination to establish regional vaccine specific immunity by injecting genital tract associated lymph nodes or inoculating the vagina mucosae topically. Compared with parenteral immunisations, these mucosal-directed interventions elicited higher levels of both B and T cell vaccine specific immune responses [8], [9].

Polymeric intravaginal rings have a long history of use in providing controlled release of small-molecule therapeutics for hormonal contraception, hormone replacement therapy, HIV prevention and other clinical indications within women's healthcare [10], [11], [12], [13]. The application of vaginal ring technology to controlled release of macromolecules, including protein antigens, is considerably more challenging owing to the thermal instability of proteins under the processing conditions commonly used to manufacture rings and the limited permeability of proteins in the polymeric materials. Nonetheless, a small number of papers have been published reporting ring technologies offering sustained/controlled release of macromolecular therapeutic agents [14], [15], [16]. We previously reported a silicone elastomer vaginal ring device comprising a one-piece ring body into which various drug-loaded inserts could be placed [15]. The body of this ring device had a similar design to that of the matrix-type dapivirine-releasing vaginal ring being developed for HIV prevention by the International Partnership for Microbicides [17], [18], [19], except for the inclusion of three small cavities for placement of the protein drug delivery inserts. With this ring design, it is possible to deliver combinations of macromolecular and small molecule therapeutic agents (e.g. HIV antigen + HIV microbicide combinations) at independently-controlled release rates. In this study, we sought to establish whether this vaginal ring device could be used to deliver recombinant HIV-1 envelope protein CN54gp140 as a vaccine cargo that could be ‘seen’ by the immune monitoring system of the female sheep genital tract. The sheep vagina and cervix are anatomically and histologically similar and of comparative dimensions to the human female genital tract and have been used to test the delivery and pharmacokinetics of a variety of *anti*-HIV retrovirals and contraceptives within vaginal ring devices [20], [21], [22]. We also examined the role of an adjuvant in eliciting immune responses, its effect on the vaginal vault and detailed the relative antigen-specific humoral immune responses in the mucosal and systemic compartments.

## 2 Results

### 2.1 Vaccine carrier system: release of vaccine antigen

We first assessed the release kinetics of the CN54gp140 vaccine antigen from covered rod-type devices comprising a hydroxypropyl methylcellulose (HPMC) + CN54gp140 ± R848 freeze-dried insert contained within a silicone elastomer tube. The recombinant HIV Envelope (Env) protein was released into 2 mL of either PBS (pH 7.2) or a simulated vaginal fluid (SVF; pH 4.2) to model release within the vaginal vault. CN54gp140 protein released into the media was quantified using a specific capture ELISA with a known concentration CN54gp140 standard curve.

**Fig. 1:**
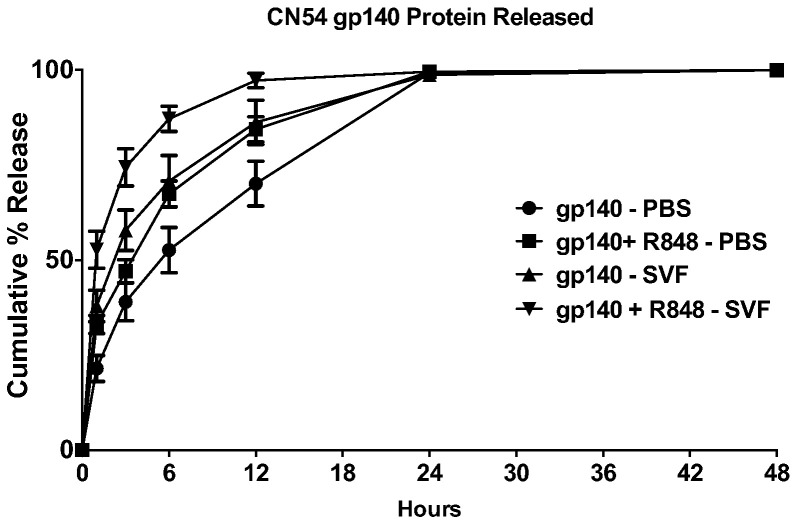
CN54gp140 in vitro release profile. Individual freeze-dried silicone elastomer rods were laced into 2 mL release media (PBS, pH 7.2 or SVF, pH 4.2). Rods contained CN54gp140 alone (167 μg) or CN54gp140 + R848 (167 μg each). The amount of CN54gp140 released into the 2 mL media was measured by quantitative ELISA and plotted as cumulative % release. Plot symbols represent the mean ± standard deviation of six replicates.

The CN54gp140 protein was released in a sustained manner over a period of 24 h, with > 99% of the total protein being released by all rod formulations in either PBS or SVF at the 24 h sample timepoint (Fig. 1). However, there were clear differences in the release kinetic depending on the nature of the release medium and whether the formulation contained R848. Freeze-dried rods released the protein more quickly into the low pH SVF media than PBS and rods containing R848 were more easily reconstituted in solution. When incubated in SVF, CN54gp140 + R848 rods released 88% of the recombinant protein within 6 h and 98% in 12 h whereas in PBS pH 7.2 67% release is achieved in 6 h and 84% in 12 h. Freeze-dried CN54gp140 alone released 71% (6 h) and 86% (12*h*) of the recombinant protein when incubated in SVF, and 53% (6 h) and 70% (12 h) when incubated in PBS pH 7.2 (Fig. 1).

### 2.2 Mixed route prime-boost combinations with TLR7/8 adjuvantation elicit high levels of CN54gp140-specific humoral immunity

**Fig. 2:**
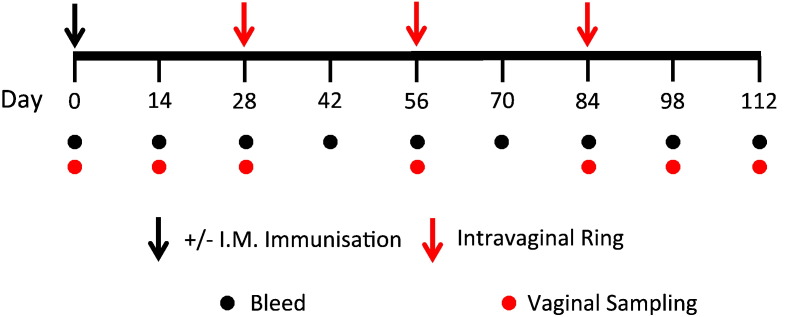
Vaccination, bleed and vaginal sampling schedule. Two-year-old sheep were immunised at monthly intervals using intravaginal rings containing CN54gp140 HIV Env antigen ± R848 adjuvant. The rings were maintained in place at the cervicovaginal area for 1 week before removal. Some sheep received a prior IM vaccination one month before the insertion of the rings. Animals were bled weekly and had vaginal mucosal antibody Weck-Cel® swabs taken at the times indicated.

We initiated a series of vaccinations to assess the ability of silicone elastomer intravaginal ring devices to deliver recombinant antigen directly to the cervicovaginal mucosa and elicit systemic or local vaccine antigen-specific humoral immunity. Sheep received either an intramuscular (IM) priming vaccination of 100 μg CN54gp140 + 200 μg R848 adjuvant followed by three intravaginal ring immunisations at monthly intervals, or intravaginal rings without any prior IM priming vaccination. The intravaginal rings all contained 500 μg recombinant CN54gp140 antigen, matched to previous intravaginal doses used in humans [23]. Two of the four animal groups received intravaginal rings that also contained the TLR7/8 adjuvant R848 (groups 1 and 2) while the other two groups received rings containing CN54gp140 antigen without any R848 adjuvantation (groups 3 and 4; Table 1). A final control group of sheep received four IM immunisations at monthly intervals (group 5; Table 1). The sheep were bled every two weeks and vaginal samples were taken at regular intervals, according to the immunisation and sampling schedule as shown (Fig. 2).

**Fig. 3:**
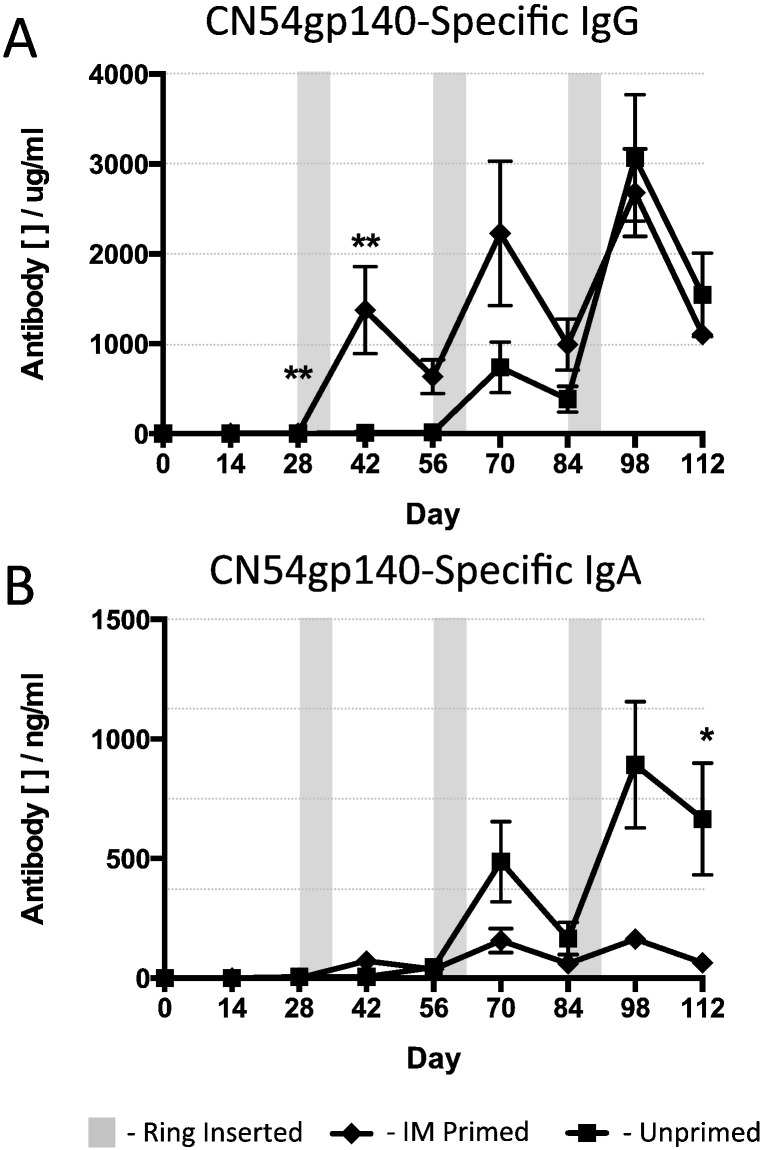
CN54gp140 antigen-specific antibody serum responses to 500 μg vaccine antigen in the presence of 500 μg R848 adjuvant per vaginal ring. IM Primed – animals received an IM prime at day 0 then three vaginal ring placements, each retained in situ for 7 days, at monthly intervals. Unprimed – animals received the vaginal rings without a prior IM vaccination. The shaded areas indicate the 7-day periods during which a ring device was present in the sheep vagina. (A) Vaccine antigen-specific IgG and (B) IgA responses. Data compared with a Mann-Whitney test (*n* = 5 per group).

Sheep that received an IM prime with adjuvant and were then boosted with an intravaginal ring device containing adjuvanted antigen exhibited strong generation of systemic antigen-specific humoral responses to the HIV Env CN54gp140 vaccine protein, increasing mean blood serum antigen-specific IgG antibody levels from 6.35 μg/mL (day 28 after IM prime) to 1375 μg/mL (day 42, two weeks after the IVag ring boost), a 216-fold increase (Fig. 3a, Table 2). Indeed, the gp140-Specific IgG responses initially elicited by the R848 adjuvanted IM injection were enhanced after each successive ring application reaching peaks of 2228 μg/mL at day 70, two weeks after the second ring application, and 2683 μg/mL at day 98, two weeks after the third and final ring was inserted. In animals that did not receive any IM prime (group 2 in Table 1) the first ring application was able to generate antigen-specific humoral immunity that was detectable in the serum. Interestingly, the second ring application substantially boosted the serum IgG gp140-specific responses by 70-fold, from 10.7 μg/mL to 739 μg/mL at the peak response two weeks after insertion of the IVag rings and the third ring application in these IM unprimed animals further boosted the serum antigen-specific IgG response to 3070 μg/mL at two-weeks post application, levels equivalent to those measured in the group 1 IM primed animals (Fig. 3a, Table 2). A comparison of the antigen-specific IgG antibody levels between the IM primed (group 1) and unprimed (group 2) animals revealed that the peak levels on day 28 and day 42 were statistically different (**p = 0.0079) with there being no difference at any other time.

The systemic antigen-specific IgA achieved significantly higher levels in animals that did not have any IM priming immunisation in comparison to those animals that had the potent systemic prime (Fig. 3b, Table 2). At the end of the vaccination schedule, following three separate intravaginal ring inoculations, the antigen-specific IgA present in the serum compartment was significantly higher than in those animals that had received an IM prime (*p = 0.0317), although levels were > 3 logs lower than that seen for specific IgG.

**Fig. 4:**
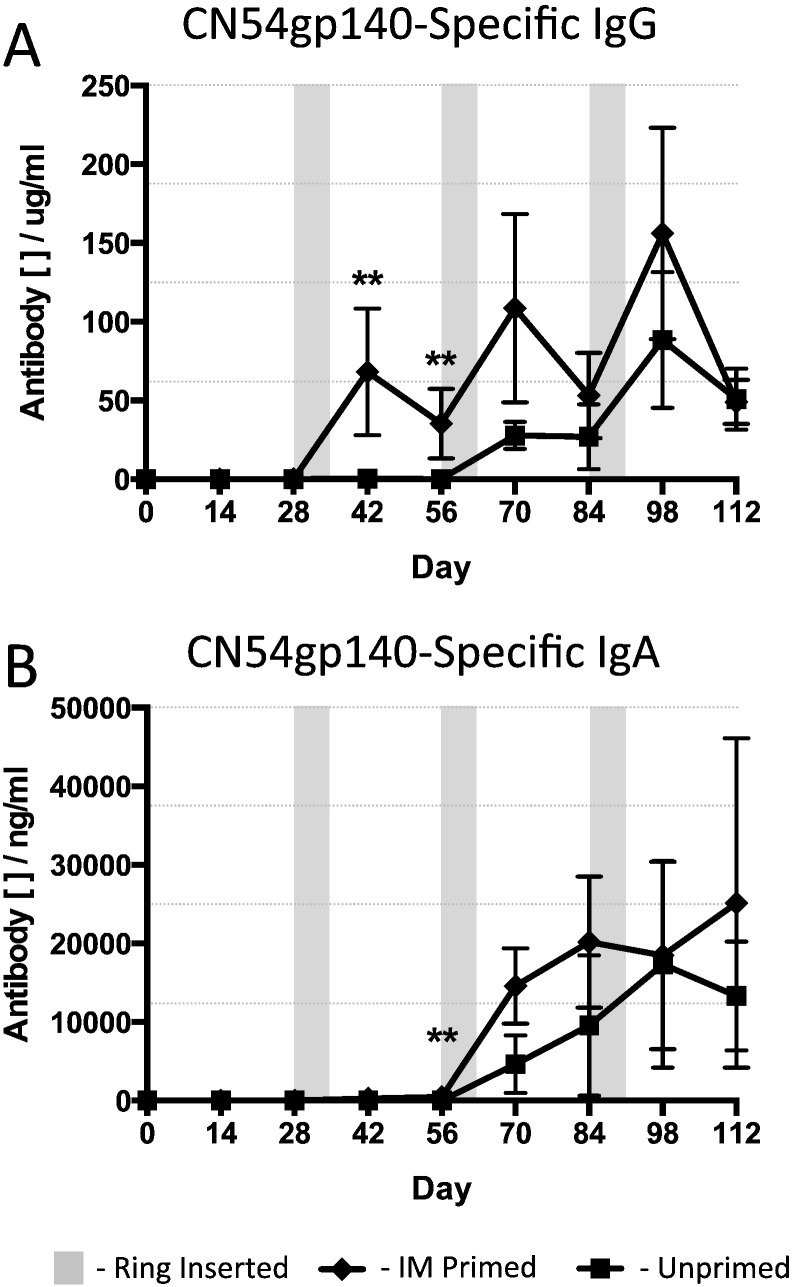
CN54gp140 antigen-specific antibody mucosal responses to 500 μg vaccine antigen in the presence of 500 μg R848 adjuvant per vaginal ring. IM Primed – animals received an IM prime at day 0 then three vaginal ring placements, each retained in situ for 7 days, at monthly intervals. Unprimed – animals received the vaginal rings without a prior IM vaccination. The shaded areas indicate the 7-day period during which a ring device was present in the sheep vagina. (A) Vaccine antigen-specific mucosal IgG and (B) mucosal IgA responses. Data compared with a Mann-Whitney test (*n* = 5 per group).

We next examined the antigen-specific immunity at the mucosal surface. We sampled the vaginal mucosae at various times during the schedule and measured gp140-specific IgG and IgA (Fig. 4). IM primed gp140-specific mucosal IgG responses were effectively enhanced by intravaginal vaccination with the ring device that contained antigen adjuvanted with R848 and antibody levels were boosted by subsequent ring applications. The specific antibody levels were statistically different between the IM primed and the unprimed groups at day 42, 2 weeks after and at day 56, one month after the insertion of the first ring (Fig. 4a; **p = 0.0079). There were no statistical differences at other sampling timepoints, and at the end of the vaccination schedule (after three intravaginal ring inoculations) the CN54gp140 antigen-specific antibody levels were equivalent (Fig. 4a, Table 2). Interestingly, antigen-specific mucosal IgA followed the same pattern. Levels were statistically different after the first ring application (day 56), where the IM primed group had higher levels in the vaginal vault, but after the second and then the final ring vaccination the levels were equivalent. Importantly, the levels of mucosal IgA were substantially higher at the mucosal surface compared to the systemic serum compartment. Animals that had not been previously primed with an IM vaccination had 20-fold higher IgA in the vaginal vault while those that had received an IM prime followed by immunisation with the vaginal rings exhibited a marked difference, with the vaginal vault having 400-fold more specific antibody when compared to the serum responses (Figs. 3b, 4b and Table 2).

### 2.3 Sheep immunised with intravaginal rings without local mucosal R848 adjuvantation fail to elicit significant CN54gp140-specific humoral immunity

**Fig. 5:**
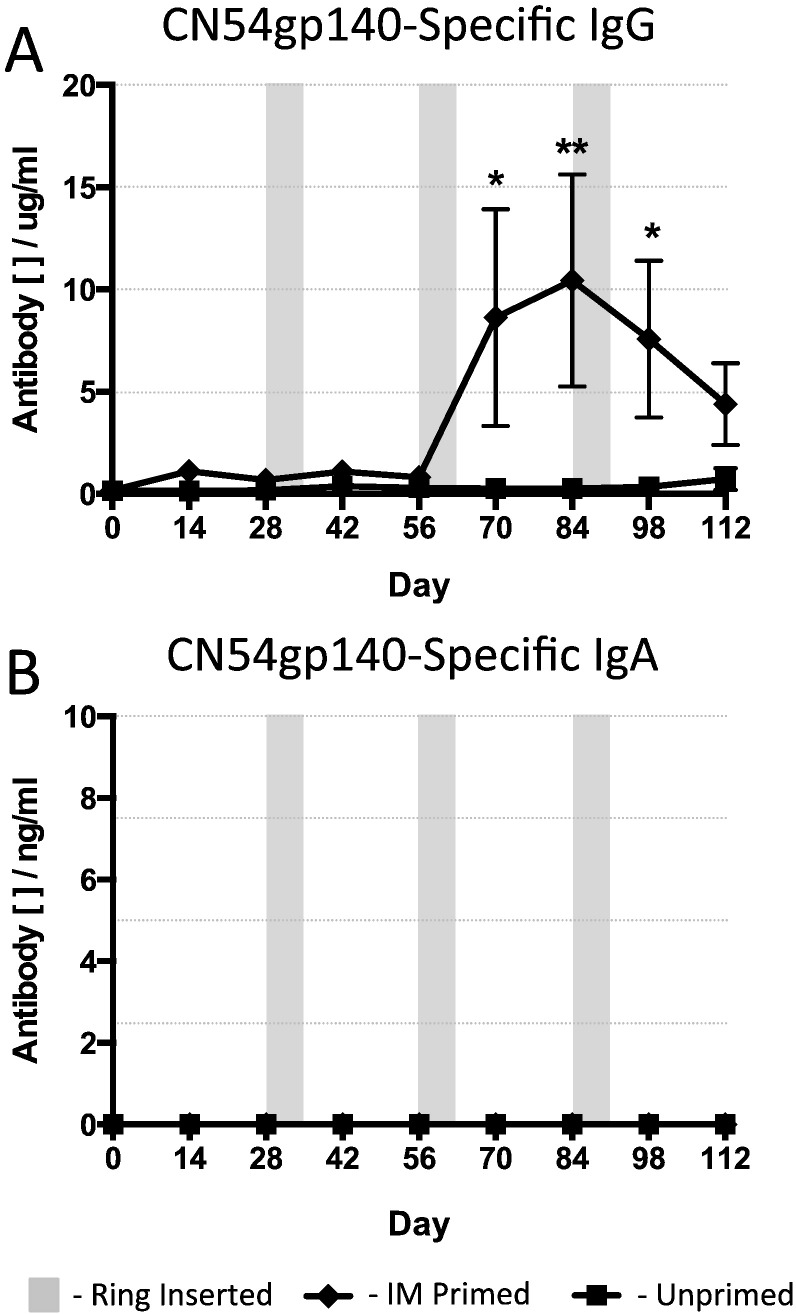
CN54gp140 antigen-specific antibody serum responses to 500 μg vaccine antigen in the absence of R848 adjuvantation. IM primed – animals received an IM prime at day 0 then three vaginal ring placements, each retained in situ for 7 days, at monthly intervals. Unprimed – animals received the vaginal rings without a prior IM vaccination. The shaded areas indicate the 7-day periods when the vaginal ring was present. (A) Vaccine antigen-specific IgG and (B) IgA responses. Data compared with a Mann-Whitney test (*n* = 5 per group).

In order to determine the contribution of the R848 TLR7/8 adjuvant to the size and kinetics of the immune response, we next examined the elicitation of antigen-specific serum and mucosal IgG and IgA in animals that were immunised with intravaginal rings that contained antigen but no R848 adjuvant. Sheep received an R848 adjuvanted IM prime and were then boosted with a vaginal ring device containing only gp140 antigen. The systemic CN54gp140-specific IgG levels were very low, though the response was enhanced after the second ring application but not after third. Animals that had not received an IM priming vaccination had statistically higher levels of mucosal IgG than primed animals at day 70 (*p* = 0.0159; two weeks after the 2nd ring insertion), day 84 (*p* = 0.0075; four weeks after the second ring insertion) and at day 98 (*p* = 0.0317; two weeks after the third ring insertion. However, peak systemic responses in these animals that did not receive the R848 adjuvant with the CN54gp140 vaccine in the ring were 300-fold lower than those seen when using rings containing R848 (Fig. 3a). In these animals no antigen-specific IgA was detected throughout the vaccination schedule (Fig. 5a, b and Table 2).

Animals that did not receive an IM prime mounted only barely detectable antigen-specific IgG responses that were 2000-fold lower than animals that also received no IM prime but were exposed to R848 adjuvanted protein within the intravaginal rings (Fig. 3, Fig. 5). There were no detectable antigen-specific IgG or IgA mucosal antibody responses in any of the animals that were vaccinated with intravaginal rings containing antigen without R848 adjuvant. At necropsy, there were no antigen-reactive cells in any of the compartments tested (Table 2).

**Fig. 6:**
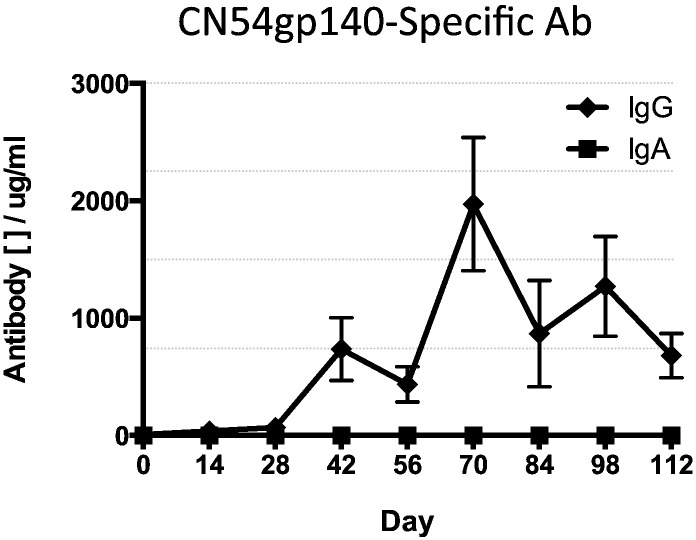
CN54gp140 antigen-specific serum antibody responses in animals that received only IM vaccinations. Animals received an IM prime of 100 μg vaccine antigen plus 200 μg R848 adjuvant at day 0 then three IM boost vaccinations with the same vaccine composition at monthly intervals. Vaccine antigen-specific IgG and IgA responses (*n* = 5 per group).

As a comparison, animals that received four IM vaccinations at monthly intervals with R848 adjuvanted CN54gp140 protein produced a good systemic IgG response reaching a maximum peak two weeks after the third vaccination with a mean of 1972 μg of CN54gp140 antigen-specific IgG (Fig. 6). The subsequent fourth vaccination failed to augment this peak response further. There was no systemic or mucosal antigen-specific IgA detected in these animals during the entire vaccination schedule (Fig. 6). Interestingly, systemic responses (IgG and IgA) were lower than those seen in animals receiving mucosal adjuvanted rings (Table 2).

**Fig. 7:**
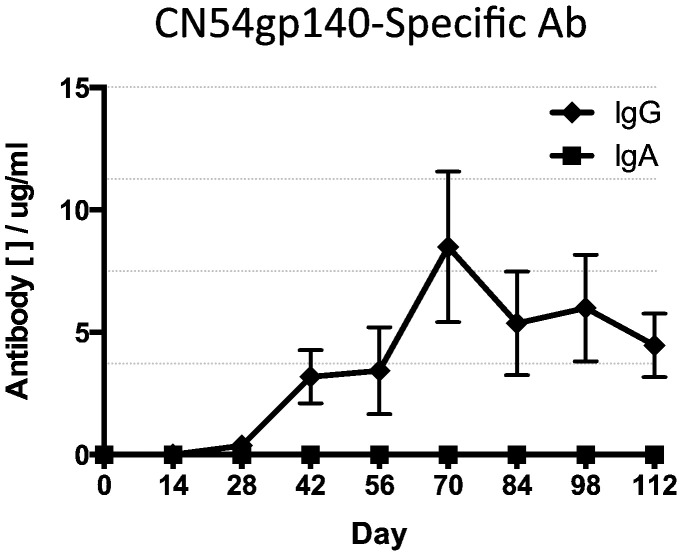
CN54gp140 antigen-specific vaginal antibody responses in animals that received only IM vaccinations. Animals received an IM prime of 100 μg vaccine antigen plus 200 μg R848 adjuvant at day 0 then three IM boost vaccinations with the same vaccine composition at monthly intervals. Vaccine antigen-specific mucosal IgG and mucosal IgA responses (*n* = 5 per group).

Mucosal antigen-specific IgG was detected at low levels in the vaginal mucosal samples and followed the profile of the systemic serum IgG precisely indicating the antibody present was derived from serum exudate (Fig. 7). Again, there was no detectable antigen-specific IgA in the vaginal samples taken throughout the vaccination regimen (Fig. 7).

### 2.4 Sheep that received intravaginal rings containing both CN54gp140 and R848 adjuvantation contained large numbers of antigen-binding cells in the local vaginal draining LN

**Fig. 8:**
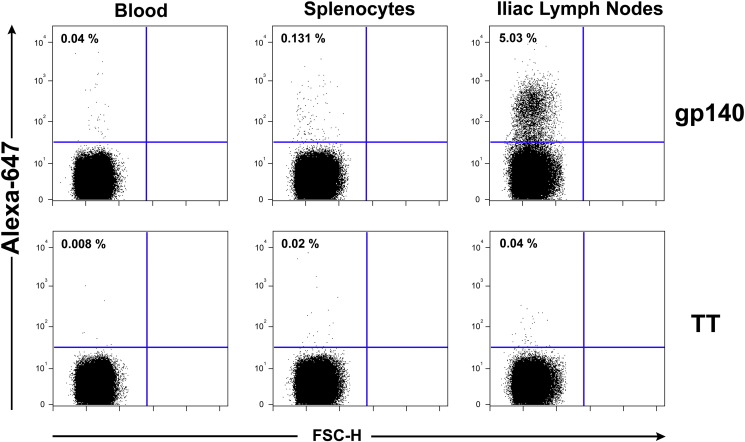
Elicitation of Antigen-Specific B cells in vaginal local draining lymph nodes. Cells were harvested from the peripheral blood, the spleen or the vaginal external iliac lymph nodes and stained with Alexa-647 conjugated recombinant CN54gp140 protein.

The notable observation that the vaginal vault contained dramatically higher levels of antigen-specific antibody in either unprimed or IM primed animals that received intravaginal rings containing CN54gp140 + R848 led us to hypothesise that this local production may be reflective of local B cell expansion and residency in the lymph nodes draining the female genital tract. At the end of the immunogenicity study, we inserted a new ring (500 μg CN54gp140 + 500 μg R848) into each sheep at day 112 before the animals were necropsied one week later. Our aim was to determine the inflammation caused by the ring after a typical 7-day application and also to examine the local lymph nodes immune response at the same time as responses in the systemic compartments. At necropsy, we removed the external iliac lymph nodes, spleen and peripheral blood from each sheep, processed to isolate lymphocytes and then incubated with a fluorochrome-labeled vaccine antigen CN54gp140-Alexa 647 or a control Tetanus Toxoid antigen (TT)-Alexa 647. B lymphocytes expressing an antigen receptor on their surface should bind the labeled antigen and the number and intensity of the bound labeled antigen positive cells was determined using flow cytometry. While the peripheral blood cells showed very low antigen-specific staining and the splenocytes demonstrated slightly more, the level of specific staining in the draining lymph nodes clearly revealed a sizeable population of cells with cell surface receptors that specifically bound the vaccine CN54gp140 antigen in comparison to the control (Fig. 8). In the absence of R848 the vaginal ring containing the unadjuvanted CN54gp140 protein did not elicit this population of antigen binding cells in the vaginal draining LN (Supplementary Material S1).

Nine out of the 10 animals that received intravaginal rings containing antigen and R848 adjuvant had large populations of antigen-specific cells in the draining external iliac lymph nodes (Table 3). We statistically compared the levels of antigen-stained cells between the blood, spleen and the vaginal draining lymph node using an unpaired *t*-tests with Welch's correction for unequal variances and found that as expected there was no difference between the levels of specific cells found in the blood and spleen for both groups of animals. However, group 1 iliac LN contained statistically higher numbers of specific cells than both the blood (*p* = 0.0207) and the spleen (*p* = 0.0225), and group 2 iliac LN also had higher numbers though only just achieving statistical differences to the blood and the spleen (*p* = 0.0499).

An important consideration when using intravaginal rings that release antigen and an adjuvant at the mucosal surface is the degree of associated inflammation. Each ring that was removed was examined for the presence of blood and none was found. At necropsy, each vagina was removed and closely examined for redness, tissue pathology and inflammation. The vaginal wall appeared normal with no apparent inflammation; a photograph showing a typical vagina with the ring still in situ is shown (Supplementary material S2).

## 3 Discussion

Conventional vaginal ring devices fabricated from hydrophobic polymers provide controlled release of low molecular actives via a molecular permeation mechanism. This involves dissolution of the drug in the surrounding polymer, diffusion of the dissolved drug molecule to the ring surface, and then partitioning into the vaginal fluid. This simple permeation mechanism is generally not useful for release of biomolecules, since their hydrophilic character and high molecular weight result in poor solubility and diffusion through the polymeric material. In the past ten years, the application of ring technology to vaginal administration of HIV microbicide molecules has led to very considerable innovation in vaginal ring design, mostly aimed at providing viable formulation solutions for lead candidate microbicides having a very broad range of physicochemical properties [10]. However, despite these advances, the development of practical ring devices that are simple and inexpensive to manufacture and can offer clinically significant drug release rates remains a challenge [16]. Here, we opted to use a ring technology previously reported for the sustained release of a model protein and a monoclonal antibody [15]. This approach permitted the silicone elastomer ring body and the antigen-loaded freeze-dried rod inserts to be manufactured separately, thereby reducing exposure of the antigen to the high processing temperatures normally associated with ring manufacture and exploiting use of established lyophilization techniques for formulation of the antigen. With this ring design, it is also possible to easily vary the dose of the antigen by reducing or increasing the number of inserts, or to incorporate an antiretroviral microbicide, such as dapivirine, into the ring body to produce a combination microbicide-vaccine formulation.

This current study examined the potential of the vaginal mucosae as a site of initiation and/or enhancement of vaccine antigen-specific immune responses. We compared a direct vaccination to a boost of an existing IM-elicited immune response using the vaginal ring delivery device, with the expectation that direct delivery of antigen to the vaginal mucosae alone might not have the potency to initiate a combined systemic and vaginal antigen-specific immune response. Surprisingly, we discovered that vaginal vaccination alone was as potent (group 2) as IM-prime, vaginal boost (group 1, Table 2), with R848 being necessary to induce high levels of antigen-specific systemic and mucosal antibody responses. Importantly vaginal vaccination with R848 was essential to induce localised mucosal responses. Further studies are now required to address the issue of dose response in relation to optimization of antigen:adjuvant ratios. Animals receiving only IM immunisation (group 5) expressed no antigen specific vaginal IgA while IgG levels were 1/10th of those seen in groups 1 and 2 (Table 2). These data are supported by the local expansion of specific B cells in lymph nodes draining the genital tract. In the absence of defined B cell markers for sheep, the nature and phenotype of local responding B and T cell responses will require further study in non-human primate and/or clinical studies.

The lack of immune response to non-adjuvanted vaginal vaccination alone and very limited response following an intramuscular prime (groups 3 and 4, Table 2) reflects human clinical studies [24], [25]. Very few clinical studies have assessed response to adjuvanted vaginal vaccination. In a previous human study using CN54gp140 and HSP70 as an adjuvant, IVag administration failed to induce detectable systemic or mucosal antibody responses but did induce adaptive CD4 and CD8 T-cell proliferative responses [9]. The difference in response may reflect difference in the adjuvant potential of HSP70 and R848, the latter associated with promoting mucosal IgA [6]. Indeed, two additional clinical studies of vaginal immunisation performed using recombinant cholera toxin subunit B (CTB), were shown to elicit vaginal and systemic antibodies [23], [26], [27]. This may reflect the “self-adjuvanting” properties of CTB, known to be an effective mucosal adjuvant for other proteins [28]. None of these studies assessed responses to vaccine delivered by vaginal rings that may offer additional advantage through direct localised delivery over topical administration of an aqueous formulation.

Both our data and previous observations in humans suggest that the inclusion of a mucosal adjuvant is likely necessary to establish effective and long-lasting mucosal immune responses. However, the downsides of using an inflammatory mediator in genital mucosal tissue are clear – the recruitment and activation of CD4 T cells has the potential to increase mucosal targets for HIV-1. Also, inflammation may compromise the integrity of the mucosal barrier increasing risk of infection not only by HIV but also other sexually transmitted infections (STIs), although an increase in mucosal expression of interferons by adjuvants may protect against pathogen infection [29], [30]. Nevertheless, this potential inflammation is likely to be short-lived. In this respect, the fact that no localised signs of inflammation were visually apparent at the end of the study is encouraging. A major advantage of the ring device is that a topical antiretroviral, such as the lead candidate microbicide dapivirine, can readily be co-delivered to the precise site of any local inflammation, protecting the mucosae during the vaccination window. In addition, the adjuvant quantity or adjuvant combinations can be tailored to maximise the immune response while preventing unnecessary bystander inflammation [2], [31], [32].

In summary, the ability to generate these vaccine-reactive B cells with local cervicovaginal adjuvanted vaccine administration using a ring release delivery system is a significant and important observation, with potential application to the future delivery modalities for any vaccine against pathogens that enter through genitourinary mucosae.

## 4 Conclusions

In this present study we have explored an intravaginal ring device for administration of a vaccine antigen at a cervicovaginal mucosal surface. We demonstrated that in the presence of a potent adjuvant a local mucosal antibody response can be generated with high levels of vaccine antigen-specific antibody and also that a population of vaccine antigen-reactive cells are established within the local draining lymph nodes, ready to respond to any re-infection with the same or similar pathogen. In this study, we used HIV Env gp140 as a model antigen but our findings are likely to be applicable to any mucosal vaccine candidate and relevant to a number of sexually transmitted diseases.

## 5 Materials and methods

### 5.1 Ethics statement

The animal studies were approved by the Ethical Review Board of St. George's, University of London where the experiments were carried out and work was performed in strict compliance with project and personal animal experimentation licences granted by the UK government in accordance with the Animals in Scientific Procedures Act (1986). Animals received minimal handling and their physical condition was monitored at least twice daily. All procedures were performed under isoflurane anaesthesia when appropriate, and all efforts were made to minimize suffering. There was a detailed protocol in place, as per requirement of the humane endpoints described in the animal licence, for early euthanasia in the event of onset of illness or significant deterioration in condition. For sheep the humane endpoints included; loss of appetite sufficient to lead to weight loss - the animals were monitored for weight weekly, loss of movement, sedentary state, calls of distress indicating pain or discomfort, bruising at site of blood withdrawal, excessive or uncontrolled bleeding from site of blood withdrawal, incontinence, breathing difficulty, infection or necrosis at site of sampling (leg vein and vaginal). The presence of one of these indicators led to an assessment by a veterinary surgeon and further welfare of the animals was directed by them. In the case of an emergency if an animal became seriously ill or injured at any point when they were on the designated premises then the animal would be first stunned by captive bolt and then killed by exsanguination before the animal regained consciousness (a non-schedule 1 method). If it were possible to handle the animal without causing it further stress and/or injury to it or staff then a schedule 1 method would be used. The captive bolt would be administered by a person licensed to use a captive bolt. One animal became ill and stopped eating during the experiment, the animal was monitored by the onsite vet, but started losing weight and exhibited a deteriorating condition. It was determined that the licence endpoint was likely to be reached and so to prevent this occurrence and any potential suffering by the animal, it was culled by the schedule 1 method of overdose of anaesthetic. Death was confirmed by cessation of blood flow. All other animals enjoyed excellent health for the duration of the experiment. At the end of the experiment all animals were culled using a schedule 1 method and death confirmed before necropsy. Food and water were supplied ad libitum.

### 5.2 Recombinant proteins and R848

HIV gp140, a trimeric gp140 clade C envelope (gp120 plus the external domain (ED) of gp41) and designated CN54gp140, was produced as a recombinant product in CHO cells and the protein manufactured to GMP specification by Polymun Scientific (Vienna, Austria). The identity of the product was confirmed by mass spectrometric analysis of tryptic fragments by the Medical Biomics Centre at St. George's, University of London. The trimeric product was stable, and has been extensively tested to validate stability even when kept at room temperature (D. Katinger - personal communication) and has previously been reported to be immunogenic. Water soluble resiquimond (R848), a low molecular weight imidazoquinoline compound was obtained from Axxora (NY, USA). MED-6382 silicone elastomer was obtained from Nusil Technology, USA. Tetrapropoxysilane (TPOS), stannous-2-ethylhexanaote (catalyst), hydroxypropyl methyl cellulose (HPMC, 6cps), were obtained from Sigma Aldrich.

### 5.3 Preparation of rings

**Fig. 9:**
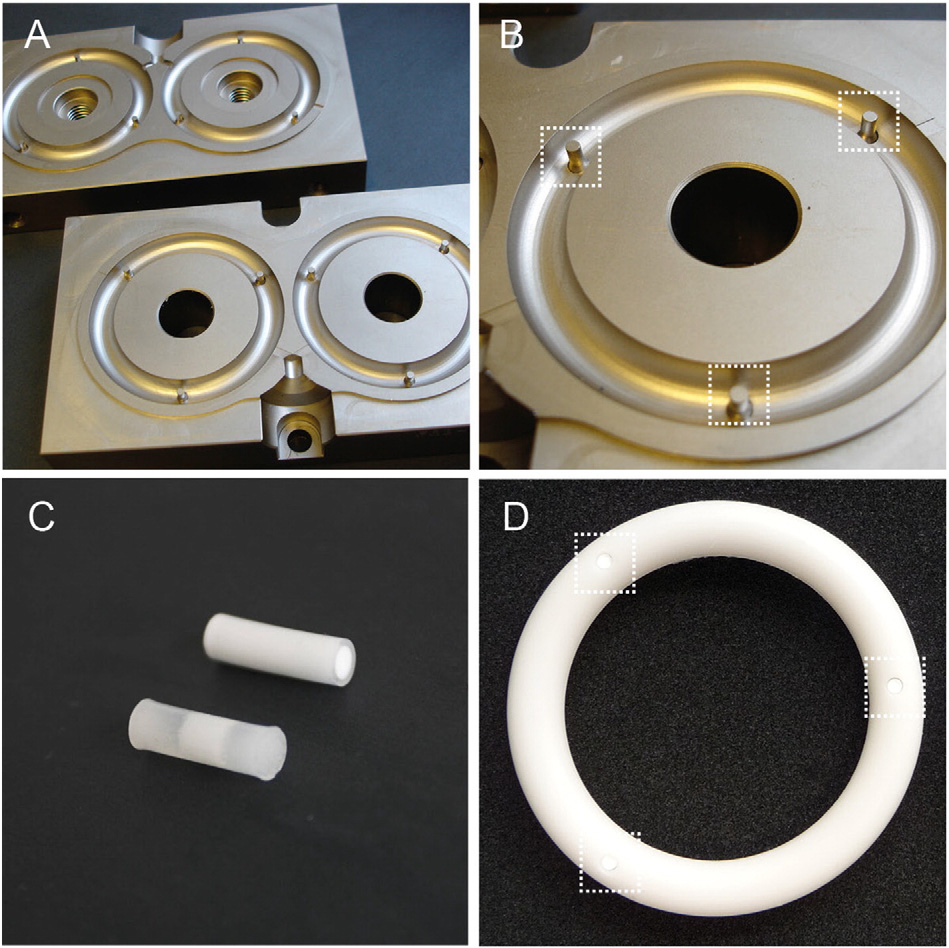
A and B – Design of injection moulds for fabrication of rod-insert vaginal ring devices. In B, the dashed boxes highlight the pins that create the cavities in the ring device. C – Examples of freeze-dried rod inserts prepared in PVC tubing. The upper rod insert is shown immediately after manufacture; the lower rod insert shows ingression of release medium. D – A silicone elastomer vaginal ring body into which three freeze-dried rod containing gp140 have been inserted.

Human sized silicone elastomer vaginal rings, containing three cavities spaced equidistantly around the ring, were prepared by elevated temperature reaction injection moulding (*T* = 80 °C, 2 min) of MED-6382 silicone elastomer mix on a laboratory-scale ring-making machine fitted with specially-designed injection moulds (Fig. 9a + b). Briefly, silicone elastomer MED-6382 was thoroughly mixed with 2.5% *w*/w TPOS using an overhead stirrer. 30 g of this mixture was mixed with 0.5% *w*/w of stannous-2-ethylhexanoate (catalyst) using a DAC 150 FVZ-K Speedmixer™ (3000 rpm, 30 s) and injected into the moulds, mounted on a custom, laboratory scale, electrically-heated injection moulding machine. The dimensions of the ring were: outer diameter: 5.8 cm, inner diameter: 4.3 cm and cross-sectional diameter: 0.76 cm.

### 5.4 Preparation of rod-inserts

Resiqimod (27.7 mg), hydroxypropyl methyl cellulose (799.8 mg), gp140 solution (2700 μL), and sterile water (473 μL) were added sequentially to a 10 mL Speedmixer™ container and mixed (60 s, 3000 rpm). The gel mixture was hydrated overnight at 2–8 °C, followed by further mixing. The gel was injected into pre-cut sections of medical-grade PVC tubing (Nalgene® metric, 7.0 mm length, 2.0 mm internal diameter, 4.0 mm outer diameter) using a 1 mL disposable plastic syringe fitted with a modified micropipette tip. Gel-filled tubes were placed on a stainless steel tray and freeze-dried (AdVantage freeze drier, VirTis, USA). The freeze drying involved ramping to − 60 °C and holding for 2 h, followed by primary drying at − 30 °C for 15 h and finally ramping to + 20 °C over 60 min and holding for 10 h [15]. After freeze-drying, the rods (Fig. 9c) were removed from the tubes and weighed. A single rod was inserted into each ring cavity (Fig. 9d). The rings were packaged in pre-labeled semi-permeable paper-plastic ring pouches and heat-sealed using a PacSeal® impulse heat sealer. Each ring device carried a total of gp140 (500 μg) and R848 (500 μg). Similar rods not containing resiqimod were also prepared by omitting its addition during preparation.

### 5.5 Release of CN54gp140 protein from silicone elastomer encapsulated rod

Individual silicone elastomer rods containing CN54gp140 protein or CN54gp140 protein plus R848 were individually placed into 15 mL polypropylene tubes with 2 mL PBS pH 7.2 or 2 mL simulated vaginal fluid (SVF) pH 4.2 [33]. Each rod contained 167 μg CN54gp140 and 167 μg R848 with *n* = 6 repeats for each rod formulation type and buffer, tubes were incubated in an orbital shaker at 37 °C, 100 rpm. The total 2 mL release media was removed with complete media replacement at the sampling timepoints 1, 3, 6, 12, 24, 48, 72, 96, 120, 144, 168 h. CN54gp140 was quantified by a quantitative direct capture ELISA. Briefly, maxisorp high binding 96-well plates were coated overnight with 10 μg/mL sheep *anti*-gp120 HIV polyclonal antibody D7234 (Aalto Bio Reagents, Ireland), blocked with 1% BSA, 0.05% Tween-20, washed and then incubated with the sample before detection with the anti-gp41 HIV human 5F3 antibody followed by anti-human IgG HRP and final development with 50 μL/well of KPL SureBlue TMB substrate (Insight Biotechnology, UK) and stopped after 5 min by adding 50 μL/well 1 M H_2_SO_4_, and the absorbance read at 450 nm on a VersaMax spectrophotometer using SoftmaxPro software. All incubations were for 1 h at 37 °C and plates washed between each step.

### 5.6 Sheep, immunisation and sampling

Female Welsh Beulah Speckled Face sheep were used in all experiments. All ewes had previously been used for breeding and were between 2 and 3 years of age. Sheep received an intramuscular injection of 100 μg HIV CN54gp140 + 200 μg R848 followed by three successive ring applications, each of one week duration and separated by one month intervals to allow development of somatically hypermutated antigen-reactive B cells in the draining lymph nodes. Each IM dose was administered in a total volume of 500 μL. Other sheep received only the ring devices without IM priming. The ring inserts contained a total of 500 μg CN54gp140 recombinant protein per 3 rods, with some rods also containing 500 μg R848 per 3 rods. 3 rods were inserted into each ring. Rings were inserted into the vagina using a medium sized speculum to open the vaginal canal and were placed around the cervical opening. Serum and vaginal mucosal fluids were sampled every two weeks and analysed by CN54gp140 ELISA. Antigen-specific cellular responses were determined at necropsy. The studies utilizing the model antigen HIV Env CN54gp140 used five animals per group.

Blood drawn from the leg vein was allowed to clot at room temperature for 30 min then centrifuged at 500 × g for 10 min and the serum removed and stored in aliquots at − 80 °C. Vaginal sampling was performed by first inserting a medium-long sized speculum into the vagina to allow access to the vaginal wall and 2 × Weck-Cel® (Beaver-Visitec International, USA) cellulose sponge spears, pre-wet with 30 μL antibody extraction buffer (2 × PBS + Protease Inhibitor cocktail) were placed into the vagina and the speculum removed. The sponges were allowed to absorb fluid within the vagina for 5 min before removal by hand and the tips cut off while being placed into the top chamber of a Spin-X column. A further 300 μL of antibody extraction buffer was added into the top chamber of the Spin-X column with the two Weck-Cel® cellulose spears and allowed to incubate at RT for 10 min. The Spin-X® column was then centrifuged at 12,000 g for 15 min to remove large debris and isolate the fluid containing the high salt eluted antibody which was then frozen at − 80 °C.

### 5.7 Antigen-specific antibody semi-quantitative ELISA

Serum and mucosal antigen-specific binding antibodies against HIV CN54gp140 recombinant protein were measured using a standardized ELISA. Maxisorp® high binding 96-well plates were coated with 50 μL recombinant proteins at 5 μg/mL in PBS overnight at 4 °C. The standard IgG or IgA immunoglobulins were coated onto the Maxisorp® plates overnight at 4 °C. Coated plates were washed three times in PBS-T before blocking with 200 μL PBS-T containing 1% bovine serum albumin for 1 h at 37 °C. After further washing, sera diluted 1/100, 1/1000 and 1/10,000 or mucosal samples diluted 1/10, 1/50 and 1/250 were added to the antigen coated wells at 50 μL/well and incubated for 1 h at 37 °C. Plates were washed four times before the addition of 100 μL of a 1/500 dilution of rabbit *anti*-sheep IgG-HRP or a 1/1100 dilution of rabbit anti-sheep IgA secondary antibody and incubated for 1 h at 37 °C. The plates were washed four times and developed with 50 μL/well of KPL SureBlue TMB substrate (Insight Biotechnology, UK). The IgA isotype, required a secondary antibody incubation step using goat anti-rabbit Ig biotin and a subsequent streptavidin-HRP (R&D systems) amplification step prior to TMB development. The reaction was stopped after 5 min by adding 50 μL/well 1 M H_2_SO_4_, and the absorbance read at 450 nm on a VersaMax™ spectrophotometer using SoftmaxPro software.

### 5.8 Flow cytometry

Cells were harvested from the animals at necropsy. External and internal iliac lymph nodes, the spleen and peripheral blood were harvested and lymphocytes isolated. Single cell suspensions of lymphocytes derived from each of these tissues were incubated with an aqua viability dye, washed 1 × with 10 volumes PBS/0.5% FBS and then incubated with 1 μg/mL Alexa 647-conjugated CN54gp140 HIV Env protein or the control 1 μg/mL Alexa 647-conjugated TT protein for 30 min at room temperature, washed 2 × with 10 volumes PBS/0.5% FBS and then resuspended to a single cell suspension in 250 μL PBS before fixation with paraformaldehyde, final concentration of 1.5%. Dead cells were gated out using the live/dead cell aqua dye and the Alexa-647 positive staining assessed on live cells.

### 5.9 Statistical analysis

The mean serum, mucosal antibody and antigen-specific flow binding data were compared using two-tailed Mann Whitney non-parametric tests or an unpaired *t*-test with Welch's correction if the data variance was not equal.

The following are the supplementary data related to this article.

**Supplementary Fig. 1:**
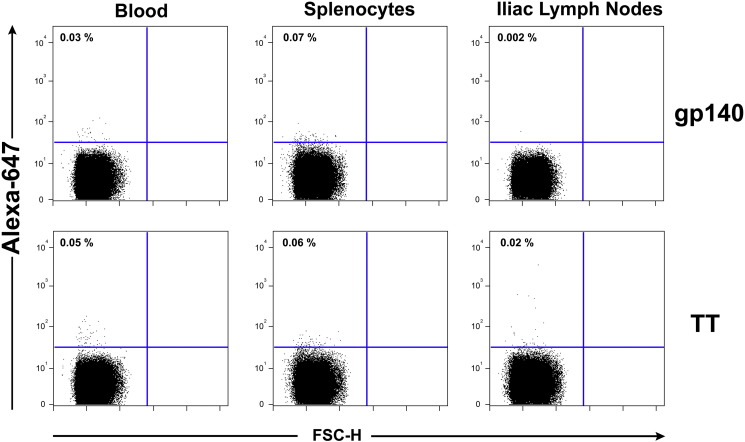
Elicitation of Antigen-Specific B cells in vaginal local draining lymph nodes of animals that received intravaginal rings containing CN54gp140 only. Cells were harvested from the peripheral blood, the spleen or the vaginal external iliac lymph nodes and stained with Alexa-647 conjugated recombinant CN54gp140 protein.

Supplementary Fig. 2 Excised sheep vagina at necropsy. A vaginal ring antigen-release device in situ within a vagina removed from a culled sheep. No inflammation or infection is apparent on the vaginal vault wall. Note that the lyophilized rod that originally contained the vaccine formulation is no longer present in the visible ring cavity. Supplementary Fig. 2

## Figures and Tables

**Table 1:** Sheep groups and immunisation schedule (*n* = 5). IM – intramuscular administration of 100 μg CN54gp140 + 200 μg R848; IVag – intravaginal administration of 500 μg CN54gp140 ± 500 μg R848.

Group	Day of Immunisation			
	0	28	56	84
1	IM	IVag/gp140 + R848	IVag/gp140 + R848	IVag/gp140 + R848
2	None	IVag/gp140 + R848	IVag/gp140 + R848	IVag/gp140 + R848
3	IM	IVag/gp140 only	IVag/gp140 only	IVag/gp140 only
4	None	IVag/gp140 only	IVag/gp140 only	IVag/gp140 only
5	IM	IM	IM	IM

**Table 2:** Serum and mucosal IgG and IgA levels (μg/mL) 2 weeks after each vaginal ring insertion (Days 42, 70 and 98) and at end of vaccination schedule (Day 112) (± SD in parentheses). IM – intramuscular administration of 100 μg CN54gp140 + 200 μg R848; IVag – intravaginal administration of 500 μg CN54gp140 ± 500 μg R848. A dash within a cell indicates that antibody levels were not detected (*n* = 5 per group).

Sheep group	Immunisation schedule*	Antibody response	Sample day			
			42	70	98	112
1	IM + 3 × IVag (gp140 + R848)	IgG systemic	1375.9 (482.6)	2228.5 (804.8)	2682.7 (488.2)	1103.9 (112.6)
		IgG mucosal	68.3 (40.2)	108.6 (53.1)	156.1 (67.0)	49.1 (14.1)
		IgA systemic	0.071 (0.024)	0.157 (0.05)	0.163 (0.061)	0.063 (0.029)
		IgA mucosal	0.286 (0.097)	14.6 (4.8)	18.5 (11.9)	25.13 (20.9)
2	3 × IVag only (gp140 + R848)	IgG systemic	10.65 (7.76)	738.4 (279.5)	3069.6 (706.2)	1543.4 (464.9)
		IgG mucosal	0.37 (0.34)	27.9 (8.6)	88.45 (43.2)	50.9 (19.3)
		IgA systemic	0.005 (0.005)	0.486 (0.167)	0.891 (0.264)	0.666 (0.234)
		IgA mucosal	– (−)	4.64 (3.7)	17.35 (13.19)	13.32 (6.93)
3	IM + 3 × IVag (gp140 only)	IgG systemic	1.08 (0.43)	8.63 (5.3)	7.57 (3.8)	4.38 (2.0)
		IgG mucosal	–	–	–	–
		IgA systemic	–	–	–	–
		IgA mucosal	–	–	–	–
4	3 × IVag only (gp140 only)	IgG systemic	0.382 (0.22)	0.255 (0.05)	0.35 (0.09)	0.726 (0.53)
		IgG mucosal	–	–	–	–
		IgA systemic	–	–	–	–
		IgA mucosal	–	–	–	–
5	IM only (gp140 + R848)	IgG systemic	735.0 (269.9)	1971.6 (568.2)	1272.1 (425.3)	680.1 (189.2)
		IgG mucosal	3.183 (1.09)	8.489 (3.07)	5.992 (2.16)	4.465 (1.3)
		IgA systemic	–	–	–	–
		IgA mucosal	–	–	–	–

**Table 3:** Mean percentage of background control subtracted antigen-specific cells in each tissue (*n* = 5 per group) (± SD in parentheses).

Sheep group	Mean % CN54gp140 specific cells		
	Blood	Spleen	Iliac LN
1	0.05 (0.012)	0.16 (0.078)	2.45 (0.802)
2	0.04 (0.014)	0.08 (0.028)	2.28 (0.839)
3	–	–	–
4	–	–	–
