# Supplementary figures and images for: Intravaginal immunisation using a novel antigen-releasing ring device elicits robust vaccine antigen-specific systemic and mucosal humoral immune responses
Source: J Control Release. 2017 Mar 10;249:74–83. doi: 10.1016/j.jconrel.2017.01.018 (PMC5333785; doi:10.1016/j.jconrel.2017.01.018)

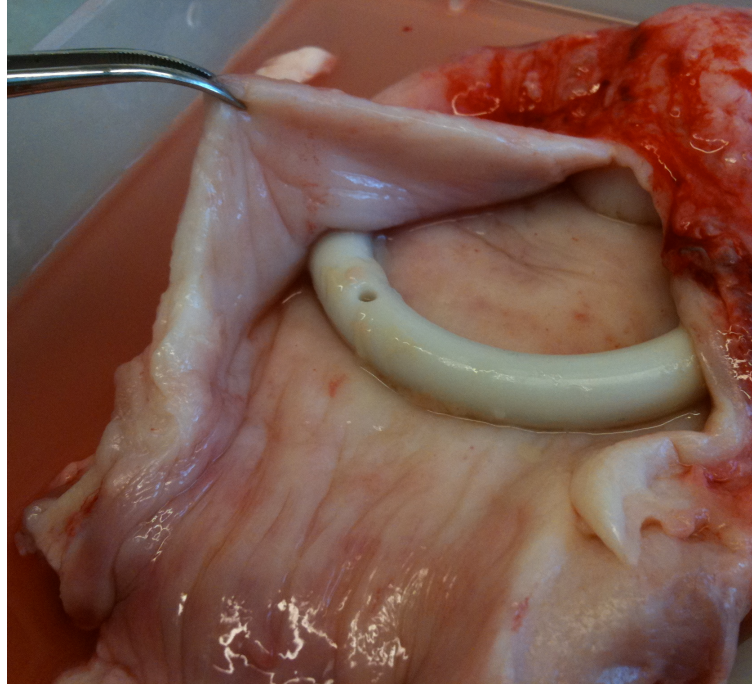

Supplement: Supplementary Fig. 2 — Excised sheep vagina at necropsy. A vaginal ring antigen-release device in situ within a vagina removed from a culled sheep. No inflammation or infection is apparent on the vaginal vault wall. Note that the lyophilized rod that originally contained the vaccine formulation is no longer present in the visible ring cavity. [file mmc1.pdf]
